# Disease Activity in Inflammatory Bowel Disease Is Associated with Increased Risk of Myocardial Infarction, Stroke and Cardiovascular Death – A Danish Nationwide Cohort Study

**DOI:** 10.1371/journal.pone.0056944

**Published:** 2013-02-15

**Authors:** Søren Lund Kristensen, Ole Ahlehoff, Jesper Lindhardsen, Rune Erichsen, Gunnar Vagn Jensen, Christian Torp-Pedersen, Ole Haagen Nielsen, Gunnar Hilmar Gislason, Peter Riis Hansen

**Affiliations:** 1 Department of Cardiology, Copenhagen University Hospital Gentofte, Hellerup, Denmark; 2 Department of Cardiology, Copenhagen University Hospital Roskilde, Roskilde, Denmark; 3 Department of Clinical Epidemiology, Aarhus University Hospital, Denmark; 4 Department of Gastroenterology, Copenhagen University Hospital Herlev, Herlev, Denmark; Universidad Peruana de Ciencias Aplicadas (UPC), Peru

## Abstract

**Purpose:**

Chronic inflammatory diseases have been linked to increased risk of atherothrombotic events, but the risk associated with inflammatory bowel disease (IBD) is unclear. We therefore examined the risk of myocardial infarction (MI), stroke, and cardiovascular death in patients with IBD.

**Methods:**

In a nationwide Danish population-based setting, a cohort of patients with incident IBD between 1996 and 2009 were identified in national registers. Hospitalizations with IBD as primary diagnosis, initiation of biological treatment and dispensed prescriptions of corticosteroids were all used as surrogate markers for disease activity, with flares classified as the first 120 days after diagnosis of IBD, and 120 days after a new corticosteroid prescription, biological treatment or IBD hospitalization, respectively. Continued corticosteroid prescriptions or IBD hospitalizations were defined as persistent activity, and periods free of such events were defined as remissions. Poisson regression was used to examine risk of MI, stroke, and cardiovascular death using a matched population-based comparison cohort as reference

**Results:**

We identified 20,795 IBD patients with a mean age of 40.3 years that were matched according to age and sex with 199,978 controls. During the study period, there were 365 patients with MI, 454 with stroke, and 778 with cardiovascular death. Patients with IBD had an overall increased risk of MI (rate ratio [RR] 1.17 [95% confidence interval 1.05–1.31]), stroke (RR 1.15 [1.04–1.27], and cardiovascular death (RR 1.35 [1.25–1.45]). During flares and persistent IBD activity the RRs of MI increased to 1.49 (1.16–1.93) and 2.05 (1.58–2.65), the RRs of stroke to 1.53 (1.22–1.92) and 1.55 (1.18–2.04) and for cardiovascular death 2.32 (2.01–2.68) and 2.50 (2.14–2.92). In remission periods, the risk of MI, stroke and cardiovascular death was similar to controls.

**Conclusion:**

Inflammatory bowel disease is associated with increased risk of MI, stroke, and cardiovascular death during periods with active disease.

## Introduction

The pivotal role of inflammatory mechanisms in the progression of atherosclerosis has fuelled research aimed at whether diseases characterized by chronic inflammation, including inflammatory bowel disease (IBD), carry an increased risk of cardiovascular disease [1], [2]. Indeed, an increased incidence of MI and stroke has been demonstrated in patients with rheumatoid arthritis, psoriasis, and systemic lupus erythematosus [3]–[5]._ENREF_3 In patients with IBD, however, studies on the risk of atherothrombotic disease are less conclusive [6]–[9]. Despite these inconclusive findings, it is well-established that patients with IBD have increased risk of developing venous thromboembolic events, and recent evidence has shown that this risk is particularly elevated during periods of increased disease activity [10], [11]. These findings are consistent with studies linking active inflammation to a general prothrombotic state [12]–[14]. IBD including the two main entities ulcerative colitis (UC) and Crohn’s disease (CD) has an estimated prevalence of 2.2 million persons in Europe alone, and linkage between IBD and atherothrombotic disease could potentially have a major impact on the management of these patients [15]. We therefore investigated the risk of MI, stroke, and cardiovascular death in patients with IBD with correlation to disease activity in a nationwide Danish cohort.

## Methods

### Data sources

The study was conducted and reported in accordance with the Strengthening the Reporting of Observational studies in Epidemiology (STROBE) recommendations [16]._ENREF_15 Each resident in Denmark is given a unique and permanent personal civil registration number at birth or immigration, which enables linkage on individual level across nationwide registers. We used information on date of birth, migration, and socioeconomic status from the civil registration system. Data on morbidity were included from the National Patient Register, holding diagnoses listed according to the international classification of diseases, 8^th^ revision (ICD-8) until 1994, and the 10^th^ revision (ICD-10) thereafter. The National Patient Register contains information on all hospital admissions (since 1978) and outpatient activities (from 1995) and at discharge each admission is registered by one primary diagnose and, if appropriate one or more secondary diagnoses [17]. The Danish Register of Medicinal Product Statistics (the national prescription register) holds complete information on all prescriptions claimed from Danish pharmacies since 1995, and each prescription is registered according to the international Anatomical Therapeutical Chemical (ATC) classification. As drug expenses in Denmark are partially reimbursed by the government-financed health care system, Danish pharmacies are required to register each dispensed prescription in the national prescription registry, which ensures complete and accurate registration [18]. Deaths are registered in the National Cause of Death Register with one primary, and if appropriate, one or more underlying or contributing causes of death. Socioeconomic status was divided into quintiles, based on mean annual taxed income in the 5 years prior to inclusion.

### Study population - Cohort entry and follow-up

In the present matched cohort study we defined IBD cases as all individuals aged ≥15 years who received a first diagnosis of IBD, i.e. CD (K50 and 563.01) or UC (K51 and 569.04+563.01), during the period 1996–2009 in combination with a dispensed prescription for pharmacological IBD treatment, including one or more of the following agents (ATC codes): 5-aminosalicylic acid (A07EC02), sulfasalazine (A07EC01), oral corticosteroids (H02AB06), budesonide (A07EA), azathioprine (L04AX01), 6-mercaptopurine (L01BB02) and methotrexate (L01BA01) within one year before the time of diagnosis and hereafter. The index dates of IBD cases were the date of IBD ICD-10 code and drug prescription, whichever came last. Initiation of biological treatment with anti-tumor necrosis factor-α (TNF) agents was defined by the Danish procedural code (BHJ18A). Surgery for IBD was defined by procedure codes (KJF [colon and small intestine], KJG and KJH [perianal area] in combination with IBD-diagnosis). The IBD diagnoses in the National Patient Registry have been found to be accurate [19]. Patients with a diagnosis of IBD, MI or stroke prior to index date were excluded. Also, individuals with a history of prescribed IBD medication (apart from corticosteroids) more than a year prior to IBD diagnosis were excluded. Each IBD patient was matched with 10 controls from the general population at time of inclusion according to age and gender. Controls with a history of MI, stroke or IBD diagnoses were excluded as well. Study subjects were followed from inclusion (index date) until MI, stroke, emigration, death or end of follow-up. Patients with a diagnosis of both UC and CD were identified as having unclassified IBD.

### IBD activity

**Figure 1:**
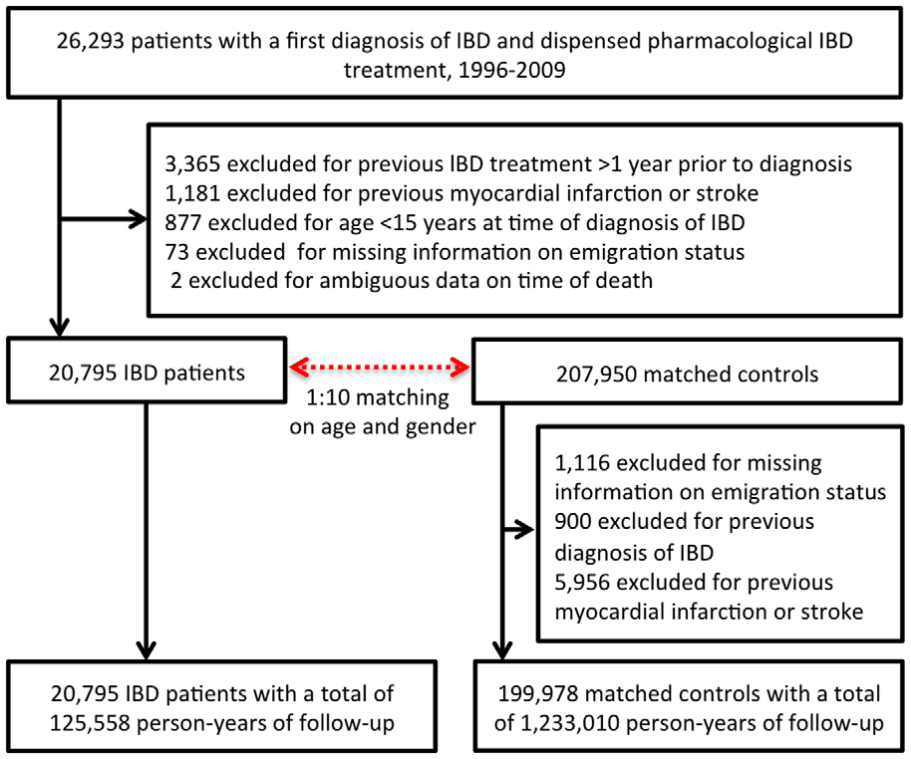
Example of disease activity periods and hospitalizations in apatient with inflammatory bowel disease (IBD) throughout the study period.

Hospitalizations with IBD as primary diagnosis, initiation of anti-TNF treatment and dispensed prescription of corticosteroids were used as surrogate markers for disease activity. An IBD flare was defined as a 120 days period starting at the day of initiation of corticosteroid treatment, biological treatment and/or hospitalization for IBD, following 120 days free of corticosteroid prescription or hospitalizations due to IBD (Fig.1) [10]. The first 120 days after study inclusion were likewise defined as a flare period. We further defined periods of persistent activity, as those which succeeded flare periods if additional hospitalizations, anti-TNF treatment or corticosteroid prescriptions had taken place within the 120 days from flare start. Remission periods started 120 days after last hospitalization, anti-TNF treatment or corticosteroid prescription and ended at the time of reinitiating of corticosteroid treatment or hospitalization. We also did sensitivity analyses where corticosteroid prescriptions were excluded as an activity marker.

### Co-morbidity and medical treatment

We assessed the presence of co-morbidity at study entry by hospitalization for the following prespecified diagnoses in the 5 years preceding study inclusion (ICD-10 and ICD-8 codes): Cardiac dysrhythmia (I44–I49 and 427.3, 427.4, 427.5, 427.6, 427.9), diabetes (E10–E14 and 250), chronic obstructive pulmonary disease (J42, J44 and 490–492), renal disease (N03, N04, N17, N18, N19, R34, I12, I13 and 582–588), hypertension (I10–I15 and 400–404), venous thromboembolic disease (I26, I80, I82 and 415, 453 excluding I80.8, I80.0 and I82.0), and heart failure (I42, I43, I50 and 110, 517)._ENREF_19Use of the following drugs (ATC codes) was defined at study inclusion : Glucose-lowering agents (A10), statins (C10A), loop diuretics (C03C), platelet inhibitors (B01AC), vitamin K antagonists (B01AA) and we identified patients with hypertension by treatment with at least two of the following classes of antihypertensive drugs: α-adrenergic blockers (C02A, C02B, C02C), non-loop diuretics (C02DA, C02L, C03A, C03B, C03D, C03E, C03X, C07C, C07D, C08G, C09BA, C09DA, C09XA52), vasodilators (C02DB, C02DD, C02DG), β blockers (C07), calcium channel blockers (C07F, C08, C09BB, C09DB), and renin-angiotensin system inhibitors (C09). Similarly we defined diabetes by either the hospital diagnosis (E10–E14) or use of glucose-lowering agents (A10). All information on cardiovascular medication and co-morbidity were continually updated throughout the follow-up period.

### Study endpoints

The following endpoints (ICD-10 codes) were used: MI (I21–I22), stroke (I60–I61, I63–I64), cardiovascular death (I00–I99) and a secondary composite endpoint of MI, stroke and cardiovascular death. The stroke and MI codes have previously been validated in the National Patient Register [20], [21].

### Statistical analysis

Baseline characteristic were summarized as means with standard deviations for continuous variables and frequencies and percentages for categorical variables. Incidence rates (IRs) are presented per 1000 person-years. To estimate rate ratios (RRs) and 95% confidence intervals (CIs) for MI, stroke, cardiovascular death, and the composite endpoint, we fitted multivariable Poisson regression models in patients with IBD using the matched controls as reference. The models were adjusted for confounding factors, including socioeconomic status, and gender. Age, co-morbidity, use of cardiovascular medication (antihypertensive treatment, statins, loop diuretics, vitamin K antagonists), lipid-lowering agents, glucose-lowering medication, and IBD disease activity status were included as time-dependent variables. Subjects lost to follow-up due to emigration from Denmark were censored at time of emigration. To address potential differences in risk of cardiovascular disease in patients with CD, UC or unspecified IBD we evaluated overall risk and disease activity related risk for each endpoint in an IBD subtype-stratified analysis. In addition, we changed the flare duration to assess the potential impact of flare-definition on the risk estimates. We did subgroup analyses of patients that received anti-TNF agents (BHJ18A) and other immunomodulators including 6-mercaptopurine (L01BA01), azathioprine (L01BB02), and/or methotrexate (L04AX). We also did a subgroup analysis where we evaluated the influence of nine predefined risk factors (prior venous thromboembolism, heart failure, cardiac arrhythmias, chronic obstructive pulmonary disease [COPD], renal disease, hypertension, diabetes, and use of loop diuretics, lipid-lowering agents, and vitamin K antagonists) and stratified all IBD patients in groups of 0 (reference group), 1–2 or ≥3 risk factors. SAS version 9.2 and Stata version 11.1 were used for statistical analyses. Risk set matching was performed with Greedy matching macro (last accessed 5 September 2012 at http://mayoresearch.mayo.edu/mayo/research/biostat/upload/gmatch.sas). We tested model assumptions, including the linearity of continuous variables and absence of interactions, and found them to be valid unless otherwise specified. Evaluation of the significance of an unmeasured confounder was made using the “rule out” approach for all reported results [22].

### Ethics

Register-based studies do not require ethical approval in Denmark as individual patients cannot be identified from the encrypted data that are available. The Danish Data protection agency approved the study (reference no. 2007-58-0015, international reference: GEH-2010-001).

## Results

**Figure 2:**
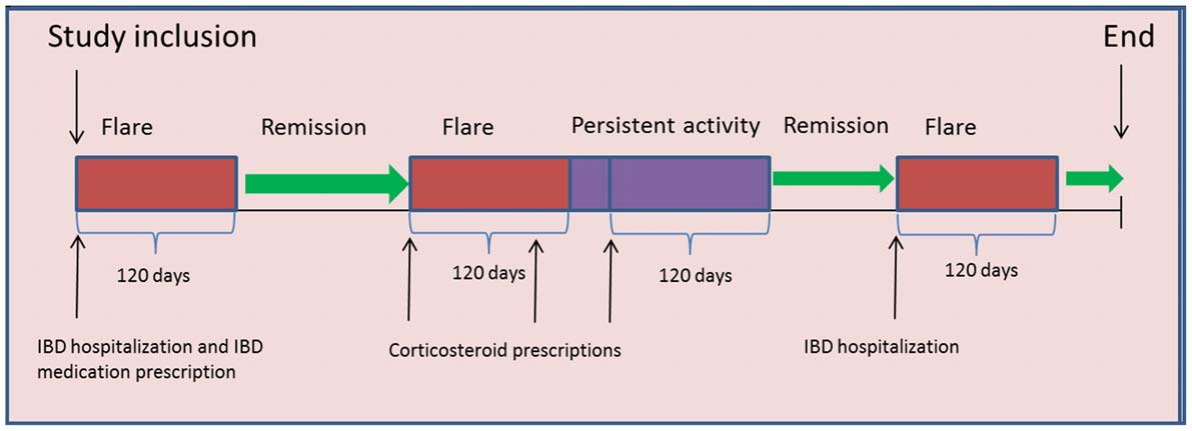
Flowchart for the study population, IBD: Inflammatory bowel disease.

A total of 26,293 IBD patients were identified with in the study period. After exclusion of patients with prior IBD, MI or stroke, the final study population included 20,795 patients (Fig. 2). A total of 199,978 matched controls were enrolled in the study.

Patient characteristics at index are displayed in Table 1. The mean age of the study population was 43.8 (SD 18.7) years, and 54.5 % were women. Loss to follow-up due to emigration was 2.0 % among the included IBD cases and 3.5 % among controls. The frequencies of co-morbidities were significantly higher among IBD patients compared to the matched controls, and use of cardiovascular drugs and glucose-lowering agents at baseline was significantly higher in the IBD group. Distribution of IBD disease activity is shown in table 2.

**Table 1:** Baseline characteristics of the study population. IBD: inflammatory bowel disease.

	IBD patients n = 20,795	Controls n = 199,978	p -value
**Male (%)**	9,455 (45.5)	90,439 (45.2)	
**Female (%)**	11,340 (54.5)	109,539 (54.8)	
**Age, years mean (SD)**	43.8 (18.7)	43.1 (18.7)	
**Mean follow-up time, years**	6.04	6.17	
**No. of patient-years**	125,558	1,233,010	
**Ulcerative colitis (%)**	13,622 (65.5)	–	
**Crohńs disease (%)**	4,732 (22.8)	–	
**Unspecific IBD (%)**	2,441 (11.7)	–	
**Treatment (%)**			
Antihypertensive treatment	1,806 (8.7)	10,362 (5.2)	<0.001
Statin	871 (4.2)	5,337 (2.7)	<0.001
Platelet inhibitor	1,222 (5.9)	6,845 (3.4)	<0.001
Loop diuretic	829 (4.0)	3,968 (2.0)	<0.001
Vitamin K antagonist	233 (1.1)	1,255 (0.6)	<0.001
Glucose-lowering medication	479 (2.3)	3,527 (1.8)	<0.001
**Co morbidity (%)**			
Cardiac dysrhythmia	409 (2.0)	1,938 (1.0)	<0.001
Heart failure	221 (1.1)	761 (0.4)	<0.001
Renal disease	102 (0.5)	275 (0.1)	<0.001
Diabetes	375 (1.8)	155 (0.1)	<0.001
Hypertension	635 (3.1)	2,335 (1.2)	<0.001
Venous thromboembolism	195 (0.9)	868 (0.4)	<0.001
Chronic obstructive pulmonary disease	403 (1.9)	1,125 (0.6)	<0.001

**Table 2:** Disease activity periods and follow-up time in study cohort with inflammatory bowel disease (IBD).

	Disease activity periods (n [%])	Mean duration (days)	Total duration of follow-up in IBD population (person-years)
**Flare**	42,685 (42.8)	117.0	13,674 (10.9)
**Persistent activity**	17,542 (17.6)	128.4	6,166 (4.9)
**Remission**	39,547 (29.1)	976.4	105,718 (84.2)
**Total**	99,774 (100)	459.6	125,558(100)

We observed a total of 365 MIs, 454 strokes and 778 cardiovascular deaths in the IBD cohort as compared to 2,389 MIs, 3,327 strokes and 4,738 cardiovascular deaths in the matched control group during follow-up.

**Figure 3:**
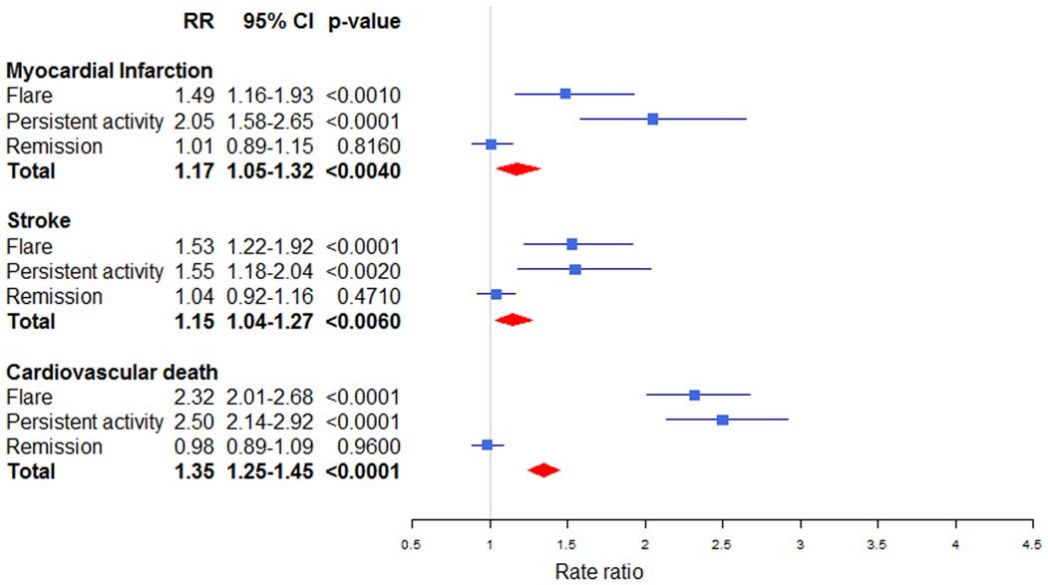
Risk of myocardial infarction, stroke and cardiovascular death stratified by inflammatory bowel disease activity. CI: confidence interval. RR: Rate ratio.

IRs for MI were 2.93 (95% CI 2.64–3.24) and 1.95 (1.87–2.03) per 1000 person-years for IBD patients and matched controls. The risk of MI was increased both in unadjusted and adjusted analyses, with an adjusted overall risk of RR 1.17 (1.05–1.31). During flares RR was 1.49 (1.16–1.93) and during persistent activity the RR was 2.05 (1.58–2.65) (Fig. 3 and Table 3). During remission the RR for MI was not increased (1.01 [0.89–1.15]) and it was significantly lower than RRs during flares (p = 0.005) and in periods with persistent activity of IBD (p<0.0001).

**Table 3:** Number of events, incidence rates per 1000 person-years, adjusted rate ratios (RRs) and 95% confidence intervals (CIs).

	Number of events	Incidence rate (unadjusted)	95% CI	RR	95% CI
**Myocardial infarction**					
Ulcerative colitis	272	3.42	3.03–3.85	1.17	1.03–1.33
Crohńs disease	61	2.16	1.68–2.78	1.35	1.03–1.77
Unspecific IBD	32	1.89	1.33–2.67	1.05	0.76–1.48
IBD total	365	2.93	2.64–3.24	1.17	1.05–1.31
Age>45 years	334	7.08	6.36–7.89	1.18	1.05–1.32
Flare	60	4.36	3.38–5.61	1.49	1.16–1.93
Persistent activity	60	9.73	7.56–12.53	2.05	1.58–2.65
Remission	245	2.34	2.06–2.65	1.01	0.89–1.15
Controls	2,389	1.95	1.87–2.03	ref.	–
**Stroke**					
Ulcerative colitis	309	3.88	3.47–4.34	1.10	1.02–1.19
Crohńs disease	89	3.17	2.57–3.90	1.37	1.10–1.72
Unspecific IBD	56	3.30	2.54–4.29	1.41	1.06–1.88
IBD total	454	3.64	3.32–3.99	1.15	1.04–1.27
Age>45 years	410	8.71	7.90–9.59	1.15	1.03–1.27
Flare	77	5.60	4.48–7.00	1.53	1.22–1.92
Persistent activity	53	8.60	6.57–11.25	1.55	1.18–2.04
Remission	324	3.09	2.77–3.45	1.04	0.92–1.16
Controls	3,327	2.72	2.62–2.81	ref.	–
**Cardiovascular death**					
Ulcerative colitis	540	6.73	6.19–7.32	1.25	1.14–1.37
Crohńs disease	148	5.23	4.45–6.14	1.63	1.36–1.95
Unspecific IBD	90	5.28	4.30–6.49	1.69	1.34–2.13
IBD total	778	6.20	5.78–6.65	1.35	1.25–1.45
Age>45 years	758	15.85	14.76–17.02	1.29	1.22–1.37
Flare	192	13.89	12.06–16.01	2.32	2.01–2.68
Persistent activity	168	26.91	23.13–31.30	2.50	2.14–2.92
Remission	418	3.96	3.60–4.36	0.98	0.89–1.09
Controls	4,738	3.84	3.73–3.95	ref.	–
**Composite endpoint**					
Ulcerative colitis	869	10.99	10.28–11.74	1.17	1.09–1.26
Crohńs disease	229	8.18	7.19–9.31	1.48	1.28–1.70
Unspecific IBD	138	8.18	6.92–9.67	1.38	1.15–1.65
IBD total	1,236	9.97	9.43–10.54	1.24	1.16–1.31
Age>45 years	1,155	24.87	23.47–26.34	1.26	1.18–1.34
Flare	266	19.41	17.22–21.89	1.97	1.74–2.22
Persistent activity	205	33.67	29.36–38.60	2.07	1.80–2.39
Remission	765	7.35	6.84–7.88	1.00	0.93–1.08
Controls	8,056	6.60	6.46–6.75	ref.	–

The composite endpoint was myocardial infarction, stroke and cardiovascular death combined. IBD: inflammatory bowel disease. The model was adjusted for age, gender, co-morbidity, cardiovascular medication and socioeconomic status.

The incidence of stroke was also highest during periods of flares (IR 5.60 [4.48–7.00]) and persistent activity (IR 8.60 [6.57–11.25]) and as compared to controls (IR 2.72 [2.62–2.81]). The overall adjusted RR for stroke was 1.15 (1.04–1.27), and the risk was evenly increased in periods of flare and persistent activity with RR 1.53 (1.22–1.92) and RR 1.55 (1.18–2.04). Again, the risk during remission was negligible with RR 1.04(0.92–1.16) and significantly lower than periods with persistent activity (p = 0.008) and flares (p = 0.003)

Concerning cardiovascular death the IRs were markedly increased during flares (IR 13.89 [12.06–16.01]) and persistent activity (IR 26.91 [23.13.31.30]) compared to remission (IR 3.96 [3.60–4.36]) and matched controls (IR 3.84 [3.73–3.95]). The augmented risk in the IBD cohort was prominent for cardiovascular death with an overall increased RR of 1.35 (1.25–1.45) in the adjusted analysis. The risk of cardiovascular death was more than two-fold increased both in periods of flares (RR 2.32 [2.01–2.68]) and periods of persistent activity (RR 2.50 [2.14–2.92]). Again, the RRs were higher for flares and periods with persistent activity in comparison with remission periods (both p<0.0001). For IBD in remission, the risk was similar to matched controls (RR 0.98 [0.89–1.09]; p = 0.96). Finally for the composite endpoint of MI, stroke, and cardiovascular death, the RR was 1.97 (1.74–2.22) during flares and 2.07 (1.80–2.39) in periods with persistent activity. Once more there was no increased risk during remission RR 1.00 (0.93–1.08).

We saw no differences in cardiovascular risk between men and women with IBD, and we observed no interaction between IBD subtypes and the cardiovascular risk stratified by disease activity. The overall risk was similar for MI and augmented for stroke and cardiovascular death in CD patients as compared to UC patients (MI: RR 1.35 [1.03–1.77] vs. 1.17 [1.03–1.33] p = 0.81, stroke: RR 1.37 [1.10–1.72] vs. 1.10 [1.02–1.19] p = 0.02 and cardiovascular death: RR 1.63 [1.36–1.95] vs. 1.25 [1.14–1.37] p = 0.04). In IBD activity analyses without corticosteroid prescriptions as activity marker, we found that the higher cardiovascular risk in periods of IBD disease activity persisted (not shown). When we removed hospitalization from our IBD disease activity definition, we found similar risks of MI (RR 1.43 [1.09–1.87] vs. 1.49 [1.16–1.93]) and stroke (RR 1.46 [1.15–1.86] vs. 1.53 [1.22–1.92]) during flares. Additionally we compared the risk 120 days after surgery due to pancolitis (K51.0) and proctitis (K51.2) in UC patients, and surgery for isolated colon disease (K50.1) versus more widespread CD disease (K50.8) in CD patients, respectively. In general, we found elevated risks during this period (all RŔs >2) but due to low number of events no significant differences were found between the aforementioned groups (not shown).

When we reduced flare length to 60 days, the risk for the composite endpoint in periods with persistent activity was RR 2.67 (2.25–3.18) and during flares RR 2.08 (1.82–2.37). Also, when flare duration was increased to 180 days the corresponding RR was 1.92 (1.68–2.20) in periods with persistent activity and RR 1.75 (1.57–1.98) during flares. We identified 679 (3.3 %) patients who received anti-TNF agents in the period from inclusion to end of study. These patients were younger (median [IQR] age 27.6 [20.7–37.6] years) and had shorter (median 1.2 years) follow up time than the general IBD cohort. We found no cardiovascular events among the patients treated with anti-TNF agents within the study period.

In total 6,017 patients (28.9 %) who received treatment with 6-mercaptopurine, azathioprine and/or methotrexate. In these subjects, we found no significant differences on the risks of MI, stroke and cardiovascular death as compared to the total IBD population (MI: RR 1.15 vs. 1.17 p = 0.88, stroke RR 1.16 vs. 1.14 p = 0.79 and cardiovascular death RR: 1.23 vs. 1.35 p = 0.33). In a sensitivity analysis where we excluded patients with COPD, we found the overall risks of the cardiovascular endpoints for IBD patients essentially unchanged (MI: RR 1.16 [1.03–1.32] vs. 1.18 [1.05–1.31]], stroke: RR 1.15 [1.04–1.27] vs. 1.15 [1.04–1.27]], and cardiovascular death: RR 1.33 [1.22–1.45] vs. 1.28 [1.18–1.38]).

When we compared IBD patients with and without present cardiovascular risk factors, particularly the risk of MI was correlated to presence of risk factors, with RRs 9.09 (7.89–10.49) and 23.14 (20.00–26.77) when comparing 0 vs. 1–2, and 0 vs. ≥3 risk factors, respectively. The corresponding RRs were somewhat lower for stroke (RR 5.29 [4.80–5.87] and RR 8.21 [7.39–9.12]) and cardiovascular death (RR 2.41 [2.24–2.60] and RR 4.36 [4.04–4.70]). IBD patients without any notable cardiovascular risk factors at the time of events comprised 9.0 % of MIs, 13.2 % of strokes, and 11.8 % of cardiovascular deaths.

## Discussion

This present study examined the incidence of MI, stroke, and cardiovascular death in a nationwide cohort of more than 20,000 patients with a new onset of IBD who were followed for a mean period of 6 years. Compared to controls, we found that the risks of MI, stroke, and cardiovascular death were significantly increased during periods of IBD activity including flares and persistent activity, whereas there was no increased risk of these adverse outcomes during periods of IBD remission. The observed IBD activity-dependent increased risk suggests that systemic inflammation contributes to the pathophysiological mechanisms leading to atherothrombosis and the results parallel recent findings of IBD activity-dependent augmented risk of venous thromboembolic disease [10], [11]. Patients with CD had a higher risk of stroke and cardiovascular death as compared to UC, but we found a parallel relative risk increase regardless of IBD entity during periods with IBD activity (flares and persistent activity).

While increased risk of venous thromboembolic events in subjects with IBD is now well-established, the risk of atherothrombotic disease including MI and stroke has been the topic of debate [6]–[12]. For example, a 2007 meta-analysis of 11 studies including roughly 14,000 IBD patients _ENREF_22_ENREF_22found no increased risk of cardiovascular mortality [23]. In line with this result, two recent registry-based studies of around 17,000 and 25,000 IBD patients, respectively, reported that the risk of MI in IBD patients was comparable to matched IBD-free controls [7], [8]. However, a Canadian study of 8,000 IBD patients showed an increased risk of ischaemic heart disease (RR 1.26 [1.11–1.44]), whereas increased risk of stroke was only significant among CD patients (RR 1.26 [1.04–1.53]) [24]. In addition, in a cohort of 8,000 patients with CD from the UK General Practice Research Database, an increased risk of stroke in patients <50 years (odds ratio 2.93 [1.44–5.98]) was observed, but no increased overall risk of stroke among older patients [6]. Moreover, a retrospective single-center cohort study of around 350 IBD patients found an increased risk of coronary artery disease [25].

The current results add considerably to the existing literature by demonstrating a significantly increased risk of MI, stroke, and cardiovascular death in a large and unselected population of patients with IBD. A novel finding was that the risk was related to IBD activity with highest risk during flares and periods of persistent activity, while in remission periods the risk of MI and stroke was only marginally increased and in the latter periods the risk of cardiovascular death was comparable to the reference population. In agreement with our results, a study from the same nationwide population published during the preparation of our manuscript also reported an increased risk of ischaemic heart disease including MI in patients with IBD, with particularly high risk in the first 3 months after IBD diagnosis and in patients with a history of treatment with oral corticosteroids [26]. Importantly, that study did not examine the risk of stroke and cardiovascular death, and did not specifically explore the risk associated with different activity of IBD as done in the present study. Moreover, the primary outcome of that study, i.e. ischaemic heart disease, has not been validated in the Danish National Patient Register. These differences notwithstanding, the results clearly suggest that the systemic inflammatory burden in subjects with IBD may be an important determinant of atherothrombotic risk. In agreement with this contention, a disease severity-dependent increased risk of MI and stroke has also been found in patients with other chronic inflammatory diseases, including rheumatoid arthritis and psoriasis [27], [28].

Atherosclerosis is a chronic inflammatory disease characterized by inflammation both in the arterial wall and systemically in the body, and atherothrombotic disease is associated with increased inflammation as exemplified by elevated levels of C-reactive protein [2], [29], [30]. Indeed, the inflammatory state involves many unspecific mechanisms including release of cytokines and other mediators (including tumor necrosis factor alpha, interleukin-1, and platelet activating factor) which may contribute to shifting the hemostatic balance towards a prothrombotic state [2]. IBD is also characterized by an inappropriate immuno-inflammatory activation, and the pathophysiological processes in the colonic wall in patients with IBD share many features with the processes in the arterial wall during progression of atherosclerosis and, ultimately, atherosclerotic plaque rupture and thrombosis [12], [31]–[35]._ENREF_21_ENREF_21 Reports that IBD is associated with subclinical atherosclerosis, including endothelial dysfunction and increased carotid intima-media thickness, together with atherogenic alterations of the lipid profile, lend additional support to the current finding of an increased risk of atherothrombotic disease in these patients [14], [34], [36], [37]. Although the increased risk of atherothrombotic disease associated with IBD activity may be explained in part by an increased inflammatory activity, other contributory mechanisms should be considered as well, e.g., increased use of corticosteroids, and susceptibility to surgical interventions and infections during periods of augmented IBD activity. Specifically, corticosteroids may have pro-thrombotic effects, but it remains controversial if use of these drugs adds to the risk of atherothrombotic disease in patients with IBD or other chronic inflammatory diseases.[38]_ENREF_30 We also made a sensitivity analysis with exclusion of COPD patients and found no alterations in the overall results. Among the patients who received TNF inhibitors within the study period, we found no cardiovascular events. Whether this was due to the anti-inflammatory effects of anti-TNF agents or caused by the low median age and short follow-up time of these patients warrants further investigations. We found similar cardiovascular risk in the 6,017 IBD patients who received treatment with azathioprine, 6-mercaptopurine, and/or methotrexate as compared to the total IBD population. Whether this result reflects that these drugs truly had no effects on cardiovascular outcomes or that such effects were confounded by the selection of patients with more severe IBD is unclear at present.

Our subgroup analysis which stratified IBD patients according to presence of cardiovascular risk factors showed a strong correlation between the number of risk factors and the risk of adverse cardiovascular endpoints. Only approximately 10 % of cardiovascular events occurred in IBD patients with no notable risk factors at the time of the event.

### Strengths and limitations

The major strength of this study was the large number of IBD patients included and the completeness of data that covered the entire population of Denmark independent of race, socioeconomic status, age, or participation in health insurance programs. Also, the IBD, MI, and stroke diagnoses have been validated in the National Patient Register, with a sensitivity of 94% for both the CD and UC diagnoses and positive predictive values of 97% and 90%, respectively, and for MI a positive predictive value for MI of 93.6 % and sensitivity of 77.6 % [19], [23]. For stroke the positive predictive value ranged from 73 % for cerebral hemorrhage to 95 % for ischaemic stroke [22]. Moreover, the number of patients identified by IBD treatment agents in combination with diagnosis of IBD used for establishing the IBD diagnosis and the observed number of incident IBD patients corresponded closely to those observed in previous Danish studies [39]. Also, we were able to adjust for concomitant cardiovascular medication and co-morbidities.

The main study limitation was inherent to the observational design which precludes conclusions on causality and allows for confounding. The higher prevalence of co-morbidities found among IBD-patients could be related, in part, to their more frequent contact to the health care system and hence increased likelihood of a receiving other diagnoses and treatment (detection bias). We lacked information on important cardiovascular risk factors, e.g. lipid levels, obesity, blood pressure, and smoking, although some of these unmeasured risk factors were adjusted for, in part, by use of time-dependent surrogates including medical treatment (e.g. statins for hyperlipidaemia and antihypertensive agents for hypertension) and diagnoses (e.g. COPD for smoking). Adjustment for socioeconomic status at baseline is also likely to have integrated factors such as obesity and smoking. In addition, detection bias may have contributed to increased prevalence of co-morbidities in IBD patients owing to more frequent medical control in these subjects. These limitations notwithstanding, our study design that focused on the importance of IBD disease activity for the cardiovascular risk is likely to have reduced the importance of confounders.

Misclassifications of risk factors such as untreated hypertension, diabetes, or dyslipidaemia may be present and result in unmeasured confounding. The definition of hypertension used has been validated in a randomly selected cohort of people from the Danish population aged ≥16 years, with a positive predictive value of 80% and specificity of 94.7 % [40].

An unmeasured confounder, must be prevalent, unevenly distributed and carry a very high risk to nullify the findings, for example the increased cardiovascular risk during flare periods. We estimated that such a confounder should have a prevalence of 20% and increase RR by a factor of >2 for MI and stroke, and >6 for cardiovascular death. Comparable estimates for hypothetical ‘rule out’ confounders were apparent for persistent activity, rendering its existence unlikely [22] . Finally, our definition of active IBD in terms of flares and persistent activity from corticosteroid prescriptions and primary IBD hospitalizations was arbitrary, as was the assumption that a flare leaves the patient at risk for 120 days. Nevertheless, although the length and duration of risk is likely to vary for each individual and more precise evaluation on a patient level would be advantageous, the 120 day period has been used earlier for assessment of the IBD activity-dependent risk of venous thromboembolic events [10]. Halving the flare duration to 60 days increased the relative risk both during flares and persistent activity, whereas a 50 % increase of flare duration to 180 days led to slightly reduced relative risks (not shown). In sensitivity analyses excluding the use of corticosteroids as an activity marker, the elevated cardiovascular risk in periods of flares persisted, which indicated some robustness in our definition of IBD activity.

## Conclusions

This nationwide study of IBD patients found a significantly increased risk of MI, stroke, and cardiovascular mortality as compared to matched controls. This risk was predominantly present in periods of IBD activity, including flares and persistent activity, whereas the risk was insignificantly raised for MI and stroke and not increased for cardiovascular death during remission disease stages. The results suggest that effective treatment of IBD aimed at disease remission may reduce cardiovascular risk in these patients, and that treatment strategies for atherothrombotic risk reduction during periods of IBD activity should be explored.

## References

[1] Ross R (1999) Atherosclerosis--an inflammatory disease. N Engl J Med 340: 115–126. 9887164 10.1056/NEJM199901143400207

[2] Hansson GK (2005) Inflammation, atherosclerosis, and coronary artery disease. N Engl J Med 352: 1685–1695. 15843671 10.1056/NEJMra043430

[3] Ahlehoff O, Gislason GH, Charlot M, Jorgensen CH, Lindhardsen J, et al (2011) Psoriasis is associated with clinically significant cardiovascular risk: a Danish nationwide cohort study. J Intern Med 270: 147–157. 21114692 10.1111/j.1365-2796.2010.02310.x

[4] Meune C, Touze E, Trinquart L, Allanore Y (2009) Trends in cardiovascular mortality in patients with rheumatoid arthritis over 50 years: a systematic review and meta-analysis of cohort studies. Rheumatology (Oxford) 48: : 1309–1313. 10.1093/rheumatology/kep252 19696061

[5] Svenungsson E, Jensen-Urstad K, Heimburger M, Silveira A, Hamsten A, et al (2001) Risk factors for cardiovascular disease in systemic lupus erythematosus. Circulation 104: 1887–1893. 11602489 10.1161/hc4101.097518

[6] Andersohn F, Waring M, Garbe E (2010) Risk of ischemic stroke in patients with Crohn's disease: a population-based nested case-control study. Inflamm Bowel Dis 16: 1387–1392. 20014016 10.1002/ibd.21187

[7] Ha C, Magowan S, Accortt NA, Chen J, Stone CD (2009) Risk of arterial thrombotic events in inflammatory bowel disease. Am J Gastroenterol 104: 1445–1451. 19491858 10.1038/ajg.2009.81

[8] Osterman MT, Yang YX, Brensinger C, Forde KA, Lichtenstein GR, et al (2011) No increased risk of myocardial infarction among patients with ulcerative colitis or Crohn's disease. Clin Gastroenterol Hepatol 9: 875–880. 21749853 10.1016/j.cgh.2011.06.032 PMC3183342

[9] Gandhi S, Narula N, Marshall JK, Farkouh M (2012) Are Patients with Inflammatory Bowel Disease at Increased Risk of Coronary Artery Disease? Am J Med. 10.1016/j.amjmed.2012.03.015 22840916

[10] Grainge MJ, West J, Card TR (2010) Venous thromboembolism during active disease and remission in inflammatory bowel disease: a cohort study. Lancet 375: 657–663. 20149425 10.1016/S0140-6736(09)61963-2

[11] Kappelman MD, Horvath-Puho E, Sandler RS, Rubin DT, Ullman TA, et al (2011) Thromboembolic risk among Danish children and adults with inflammatory bowel diseases: a population-based nationwide study. Gut 60: 937–943. 21339206 10.1136/gut.2010.228585

[12] Zitomersky NL, Verhave M, Trenor CC, 3rd (2011) Thrombosis and inflammatory bowel disease: a call for improved awareness and prevention. Inflamm Bowel Dis 17: 458–470. 20848518 10.1002/ibd.21334

[13] Ageno W, Dentali F (2008) Venous thromboembolism and arterial thromboembolism. Many similarities, far beyond thrombosis per se. Thromb Haemost 100: 181–183. 18690335

[14] Gresele P, Momi S, Migliacci R (2010) Endothelium, venous thromboembolism and ischaemic cardiovascular events. Thromb Haemost 103: 56–61. 20062938 10.1160/TH09-08-0562

[15] Loftus EV Jr (2004) Clinical epidemiology of inflammatory bowel disease: Incidence, prevalence, and environmental influences. Gastroenterology 126: 1504–1517. 15168363 10.1053/j.gastro.2004.01.063

[16] von Elm E, Altman DG, Egger M, Pocock SJ, Gotzsche PC, et al (2007) The Strengthening the Reporting of Observational Studies in Epidemiology (STROBE) statement: guidelines for reporting observational studies. Lancet 370: 1453–1457. 18064739 10.1016/S0140-6736(07)61602-X

[17] Kildemoes HW, Sorensen HT, Hallas J (2011) The Danish National Prescription Registry. Scand J Public Health 39: 38–41. 21775349 10.1177/1403494810394717

[18] Gaist D, Sorensen HT, Hallas J (1997) The Danish prescription registries. Dan Med Bull 44: 445–448. 9377907

[19] Fonager K, Sorensen HT, Rasmussen SN, Moller-Petersen J, Vyberg M (1996) Assessment of the diagnoses of Crohn's disease and ulcerative colitis in a Danish hospital information system. Scand J Gastroenterol 31: 154–159. 8658038 10.3109/00365529609031980

[20] Krarup LH, Boysen G, Janjua H, Prescott E, Truelsen T (2007) Validity of stroke diagnoses in a National Register of Patients. Neuroepidemiology 28: 150–154. 17478969 10.1159/000102143

[21] Madsen M, Davidsen M, Rasmussen S, Abildstrom SZ, Osler M (2003) The validity of the diagnosis of acute myocardial infarction in routine statistics: a comparison of mortality and hospital discharge data with the Danish MONICA registry. J Clin Epidemiol 56: 124–130. 12654406 10.1016/s0895-4356(02)00591-7

[22] Schneeweiss S (2006) Sensitivity analysis and external adjustment for unmeasured confounders in epidemiologic database studies of therapeutics. Pharmacoepidemiol Drug Saf 15: 291–303. 16447304 10.1002/pds.1200

[23] Dorn SD, Sandler RS (2007) Inflammatory bowel disease is not a risk factor for cardiovascular disease mortality: results from a systematic review and meta-analysis. Am J Gastroenterol 102: 662–667. 17156143 10.1111/j.1572-0241.2006.01018.x

[24] Bernstein CN, Wajda A, Blanchard JF (2008) The incidence of arterial thromboembolic diseases in inflammatory bowel disease: a population-based study. Clin Gastroenterol Hepatol 6: 41–45. 18063423 10.1016/j.cgh.2007.09.016

[25] Yarur AJ, Deshpande AR, Pechman DM, Tamariz L, Abreu MT, et al (2011) Inflammatory bowel disease is associated with an increased incidence of cardiovascular events. Am J Gastroenterol 106: 741–747. 21386828 10.1038/ajg.2011.63

[26] Rungoe C, Basit S, Ranthe MF, Wohlfahrt J, Langholz E, et al. (2012) Risk of ischaemic heart disease in patients with inflammatory bowel disease: a nationwide Danish cohort study. Gut. 10.1136/gutjnl-2012-303285 22961677

[27] Solomon DH, Kremer J, Curtis JR, Hochberg MC, Reed G, et al (2010) Explaining the cardiovascular risk associated with rheumatoid arthritis: traditional risk factors versus markers of rheumatoid arthritis severity. Ann Rheum Dis 69: 1920–1925. 20444756 10.1136/ard.2009.122226 PMC2963658

[28] Ahlehoff O, Gislason GH, Jorgensen CH, Lindhardsen J, Charlot M, et al (2012) Psoriasis and risk of atrial fibrillation and ischaemic stroke: a Danish Nationwide Cohort Study. Eur Heart J 33: 2054–2064. 21840930 10.1093/eurheartj/ehr285

[29] Libby P, Ridker PM, Hansson GK (2009) Inflammation in atherosclerosis: from pathophysiology to practice. J Am Coll Cardiol 54: 2129–2138. 19942084 10.1016/j.jacc.2009.09.009 PMC2834169

[30] Kaptoge S, Di Angelantonio E, Lowe G, Pepys MB, Thompson SG, et al (2010) C-reactive protein concentration and risk of coronary heart disease, stroke, and mortality: an individual participant meta-analysis. Lancet 375: 132–140. 20031199 10.1016/S0140-6736(09)61717-7 PMC3162187

[31] Baumgart DC, Carding SR (2007) Inflammatory bowel disease: cause and immunobiology. Lancet 369: 1627–1640. 17499605 10.1016/S0140-6736(07)60750-8

[32] Irving PM, Pasi KJ, Rampton DS (2005) Thrombosis and inflammatory bowel disease. Clin Gastroenterol Hepatol 3: 617–628. 16206491 10.1016/s1542-3565(05)00154-0

[33] Abraham C, Cho JH (2009) Inflammatory bowel disease. N Engl J Med 361: 2066–2078. 19923578 10.1056/NEJMra0804647 PMC3491806

[34] van Leuven SI, Hezemans R, Levels JH, Snoek S, Stokkers PC, et al (2007) Enhanced atherogenesis and altered high density lipoprotein in patients with Crohn's disease. J Lipid Res 48: 2640–2646. 17890779 10.1194/jlr.M700176-JLR200

[35] Strober W, Fuss IJ (2011) Proinflammatory cytokines in the pathogenesis of inflammatory bowel diseases. Gastroenterology 140: 1756–1767. 21530742 10.1053/j.gastro.2011.02.016 PMC3773507

[36] Roifman I, Sun YC, Fedwick JP, Panaccione R, Buret AG, et al (2009) Evidence of endothelial dysfunction in patients with inflammatory bowel disease. Clin Gastroenterol Hepatol 7: 175–182. 19121648 10.1016/j.cgh.2008.10.021

[37] Dagli N, Poyrazoglu OK, Dagli AF, Sahbaz F, Karaca I, et al (2010) Is inflammatory bowel disease a risk factor for early atherosclerosis? Angiology 61: 198–204. 19398421 10.1177/0003319709333869

[38] Walker BR (2007) Glucocorticoids and cardiovascular disease. Eur J Endocrinol 157: 545–559. 17984234 10.1530/EJE-07-0455

[39] Vind I, Riis L, Jess T, Knudsen E, Pedersen N, et al (2006) Increasing incidences of inflammatory bowel disease and decreasing surgery rates in Copenhagen City and County, 2003–2005: a population-based study from the Danish Crohn colitis database. Am J Gastroenterol 101: 1274–1282. 16771949 10.1111/j.1572-0241.2006.00552.x

[40] Olesen JB, Lip GY, Hansen ML, Hansen PR, Tolstrup JS, et al (2011) Validation of risk stratification schemes for predicting stroke and thromboembolism in patients with atrial fibrillation: nationwide cohort study. BMJ 342: d124. 21282258 10.1136/bmj.d124 PMC3031123

