# Correction: Disease Activity in Inflammatory Bowel Disease Is Associated with Increased Risk of Myocardial Infarction, Stroke and Cardiovascular Death – A Danish Nationwide Cohort Study

**DOI:** 10.1371/annotation/b4a49855-87b9-436a-a4bd-bc64b50a6c93

**Published:** 2013-04-29

**Authors:** Søren Lund Kristensen, Ole Ahlehoff, Jesper Lindhardsen, Rune Erichsen, Gunnar Vagn Jensen, Christian Torp-Pedersen, Ole Haagen Nielsen, Gunnar Hilmar Gislason, Peter Riis Hansen

There were errors in Figures 1 and 2 - the figures were reversed, while the legends are correct. The correct Figures are:

Figure 1: 

**Figure:**
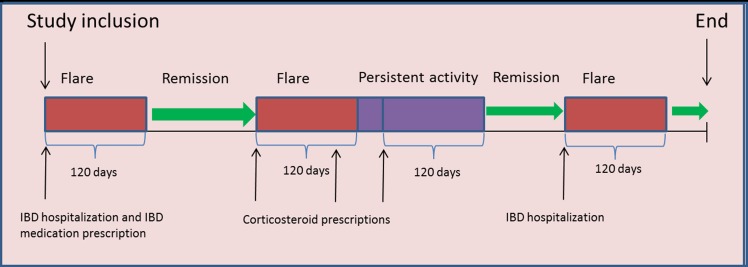


Figure 2: 

**Figure:**
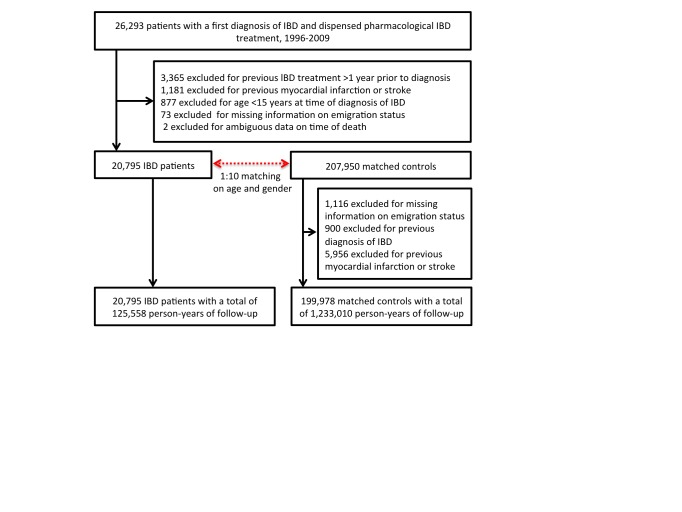


In addition, there are numerous places in the article where the typesetting process incorrectly resulted in the the text "_ENREF_#" being added to the text. 

